# Utility of interventional endoscopic ultrasound in pancreatic cancer

**DOI:** 10.3389/fonc.2023.1252824

**Published:** 2023-09-15

**Authors:** Wei On, Wafaa Ahmed, Simon Everett, Matthew Huggett, Bharat Paranandi

**Affiliations:** Department of Gastroenterology, Leeds Teaching Hospitals NHS Trust, Leeds, United Kingdom

**Keywords:** pancreatic cancer, pancreatic ductal adenocarcinoma, endoscopic ultrasound, interventional endoscopy, pancreatobiliary endoscopy

## Abstract

Endoscopic ultrasound (EUS) has an important role in the management algorithm of patients with pancreatic ductal adenocarcinoma (PDAC), typically for its diagnostic utilities. The past two decades have seen a rapid expansion of the therapeutic capabilities of EUS. Interventional EUS is now one of the more exciting developments within the field of endoscopy. The local effects of PDAC tend to be in anatomical areas which are difficult to target and endoscopy has cemented itself as a key role in managing the clinical sequelae of PDAC. Interventional EUS is increasingly utilized in situations whereby conventional endoscopy is either impossible to perform or unsuccessful. It also adds a different dimension to the host of oncological and surgical treatments for patients with PDAC. In this review, we aim to summarize the various ways in which interventional EUS could benefit patients with PDAC and aim to provide a balanced commentary on the current evidence of interventional EUS in the literature.

## 1 Introduction

Pancreatic ductal adenocarcinoma (PDAC) is a disease with a poor prognosis and an estimated 5-year survival rate of <10% (1). Survival trends have remained static over time in comparison with other forms of cancer (2). The recent Bratislava Statement highlights PDAC as part of a group of ‘neglected cancers’ due to the lack of effective treatments and visible research efforts in the understanding and treatment of the disease (3). This has galvanized position papers and statements in eminent publications to place PDAC in the spotlight as a disease in need of concentrated research in a bid to improve outcomes (4, 5). Undoubtedly, PDAC is a disease that requires a multi-faceted approach including surgeons, oncologists and endoscopists amongst other highly valued professionals. Therefore, there are multiple avenues for research and innovation in PDAC that will require coordinated involvement of separate disciplines.

Endoscopic ultrasound (EUS) was developed in the 1980s. It permits sonographic visualization of the linings of the digestive tract and the solid organs beyond it. Evaluation of the entire pancreatobiliary system from the upper digestive tract is possible. Since its inception, EUS has established itself as an invaluable and complementary tool in the diagnosis (including tissue acquisition) and staging of PDAC, alongside other imaging modalities (6, 7). Locoregional staging information obtained via EUS is often used in conjunction with other modalities to finalize decision making regarding suitability of resection.

Over the past decade, innovation and technological advances have led to endoscopists pushing the boundaries of EUS, unlocking its potential to establish it as an interventional tool for various clinical indications (8). EUS has the unique ability of accessing difficult to reach areas within the body in a minimally invasive fashion. This is particularly advantageous in treating and managing sequelae of PDAC given the deep location of the pancreas.

In this review, we aim to provide a balanced overview of interventional EUS, and highlight areas of potential future research.

## 2 EUS-guided interventions in pancreatic cancer

### 2.1 EUS-guided biliary drainage

Over the past decade, there has been widespread, international adoption of therapeutic EUS. With regards to biliary drainage, EUS allows the operator to sonographically identify and visualize the entire biliary tract. As such, this opens up an avenue for which the biliary tract can be accessed for purposes of intervention and decompression in patients with biliary obstruction. The first reported case of endoscopic ultrasound guided biliary drainage (EUS-BD) was by Giovannini, et al. in 2001 (9). They described the use of directly accessing the biliary tract from the duodenum with the use of a needle-knife (an accessory that applies diathermy to cut through tissue) under EUS guidance, with subsequent placement of a plastic stent into bile duct, effectively creating a choledocho-duodenal tract.

Since then, EUS-BD has evolved significantly and has become more sophisticated in terms of expanding the possible routes of access into the biliary tract via the development of dedicated accessories. Nowadays, EUS-BD is an umbrella term for various techniques which be broadly classified into two main routes: transmural drainage via creation of an extra-anatomical tract with a stent or via the transpapillary route with a rendezvous technique or an antegrade approach (10, 11). The in-depth technical descriptions of each approach is outside the scope of this review but it is important to be aware of the different options in EUS-BD which are available (12).

#### 2.1.1 EUS guided biliary drainage in patients with pancreatic cancer

Obstructive jaundice is a common presentation throughout the course of the disease length in patients with PDAC. Biliary decompression via endoscopic retrograde cholangiopancreatography (ERCP) is considered the first line treatment in these patients (13). However, ERCP may be unsuccessful in up to 15% of patients for a variety of reasons, which include failure to cannulate the biliary tract, surgically altered anatomy, duodenal stenosis and malignant infiltration of the papilla (14, 15). Historically, percutaneous transhepatic biliary drainage (PTBD) or surgical bypass were alternative approaches following unsuccessful ERCP. PTBD is more likely to be utilized as a rescue procedure, however, there is a high likelihood of morbidity relating to adverse events, pain, multiple re-interventions and a detrimental impact on quality of life (QOL) (16–18). A recent network meta-analysis comparing rescue procedures after ERCP failure in patients with malignant distal biliary obstruction (MDBO) demonstrated similar technical and clinical successes between EUS-BD, surgical bypass and PTBD; although there was a trend towards higher adverse events in the PTBD group (19).

Both transmural and transpapillary routes of drainage are viable options for biliary decompression in jaundiced patients with PDAC. However, the transpapillary route requires the operator to negotiate a guidewire across the malignant stenosis which can prove challenging and time-consuming. Furthermore, transpapillary drainage via the rendezvous route would be impossible in certain patients such as those with concomitant malignant duodenal stenosis. As such, the transmural route is preferred in these cases.

**Figure 1:**
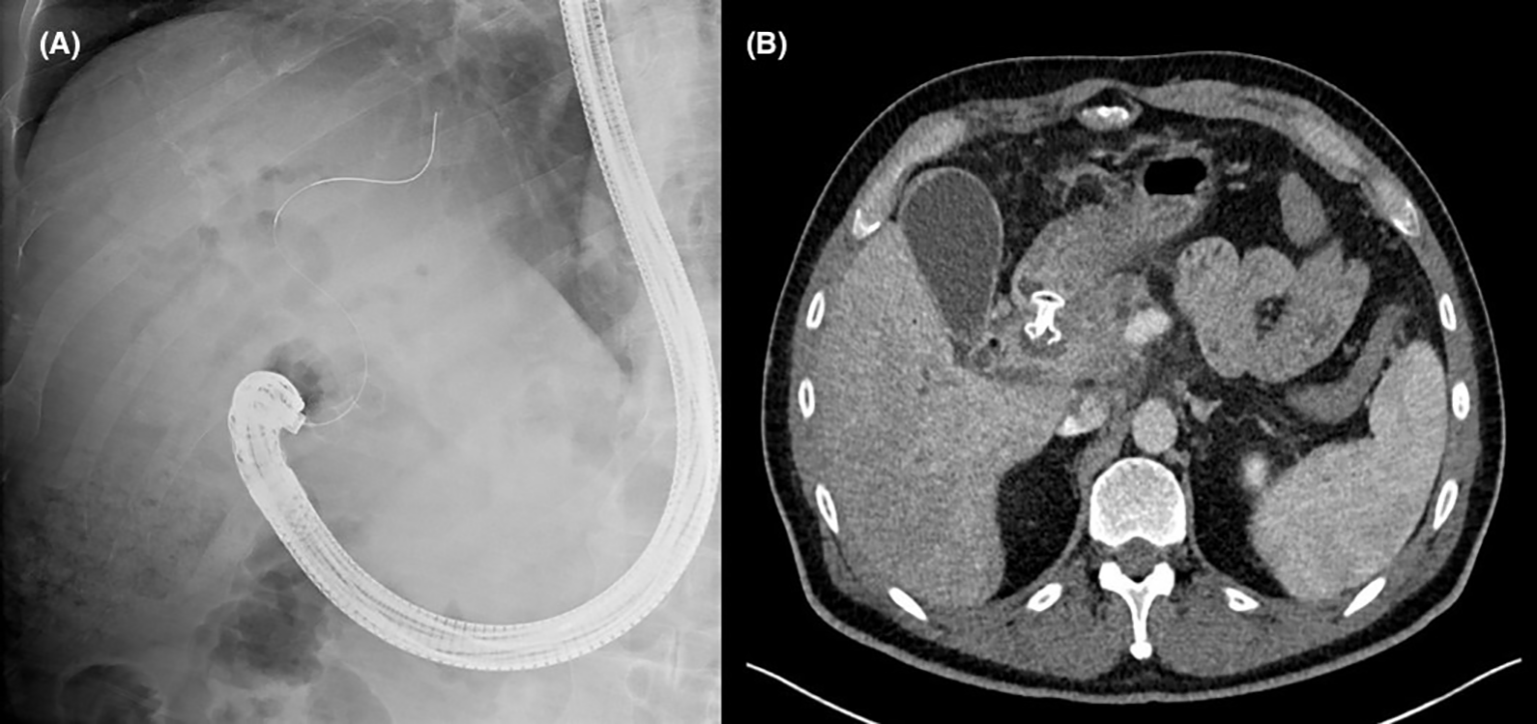
**(A)** Fluoroscopic image demonstrating successful creation of a choledochoduodenostomy with a lumen apposing metal stent. **(B)** Axial computed tomography image of the same patient showing the position of an appropriately placed choledochoduodenostomy stent.

**Figure 2:**
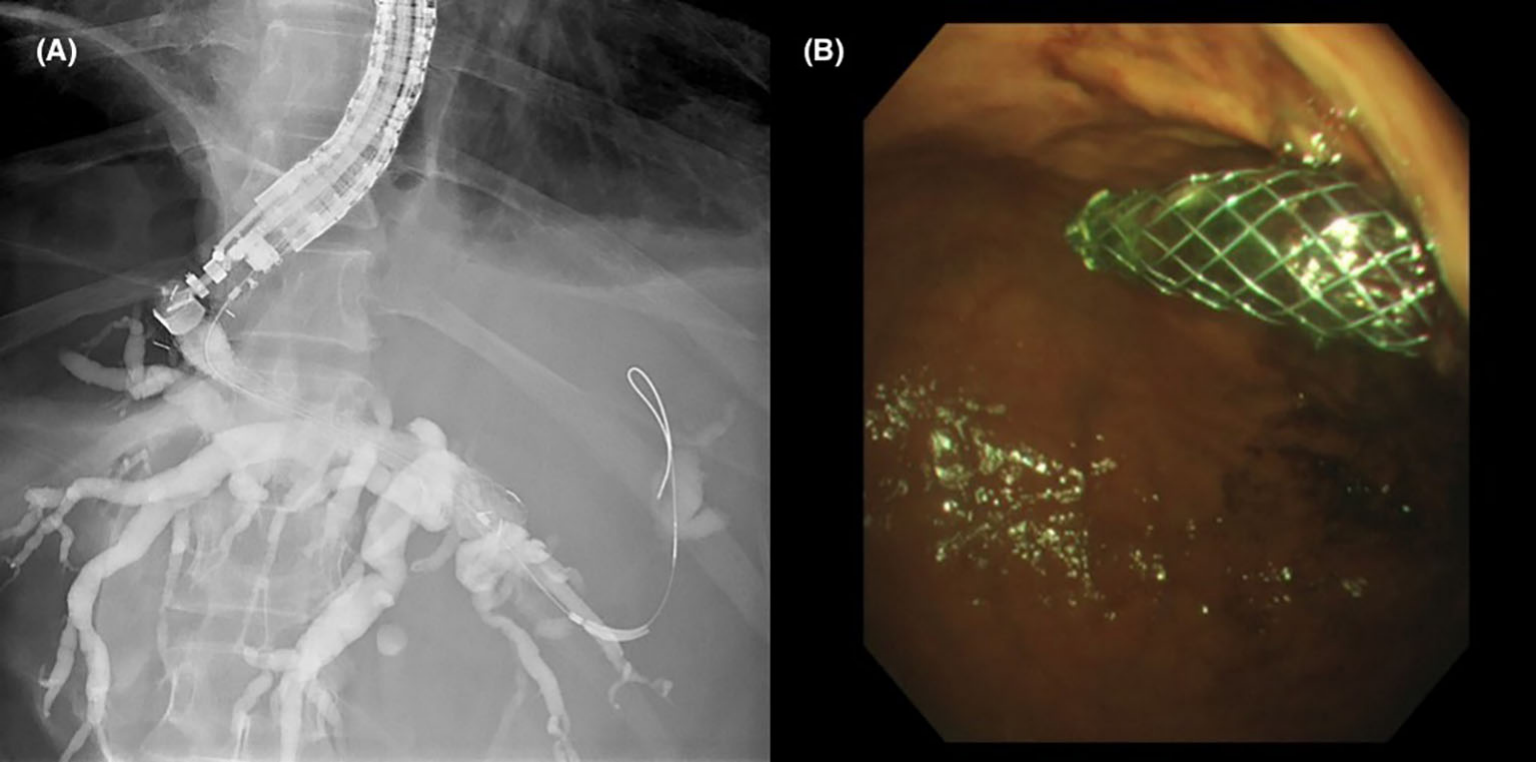
**(A)** Fluoroscopic image of contrast opacifying the left sided intrahepatic ducts with placement of a hepaticogastrostomy stent. **(B)** Gastric end of the stent protruding from the cardia into the gastric lumen.

There are two main options for transmural drainage via EUS-BD: placement of a plastic or metal stent after forming a conduit between the extrahepatic bile duct and the duodenum (**Figure 1**. choledochoduodenostomy, or CDD), or a conduit between the intrahepatic left sided bile ducts and the stomach (**Figure 2**. hepaticogastrostomy, or HGS). In patients with PDAC, EUS-CDD is preferable. This is because the level of malignant obstruction is in the distal biliary tree, therefore the close proximity of the proximally dilated extrahepatic bile duct to the duodenal wall with its relatively fixed position in the retroperitoneum renders access more straightforward (20). However, there may be some patients in whom EUS-CDD is not technically feasible; for example a lack of endoscopic duodenal access, an inadequately dilated bile duct or with paraduodenal varices precluding a safe window for access into the bile duct. In these situations, EUS-HG may be preferable as long as there is no disease affecting the plane between the left lobe of the liver and the stomach. Nevertheless, both options are viable in their respective individual circumstances, and in expert hands, are similar in terms of safety and success (21).

These techniques require multiple steps and accessory exchanges which can be time consuming, potentially increasing the risk of adverse events such as bile leakage and pneumoperitoneum (22, 23). Lumen apposing metal stents (LAMS) have gained popularity in recent times due to their versatility in various innovative applications within the digestive tract, and also particularly the relative ease and speed of deployment as a single step device. LAMS was initially developed for drainage of pancreatic fluid collections (24) but has rapidly cemented its position as the device of choice for EUS-BD in patients with MDBO. The saddle-shaped, biflanged design of the stent permits apposition between the bile duct and the duodenum as an anti-migratory measure whilst the mesh of the stent is covered to prevent bile leakage. Due to its unique design, it can only be deployed via the CDD route. The popularity of EUS-CDD with a LAMS has led to an influx of large case series in the literature from various geographical regions across the world and this route of biliary drainage has become the first choice in patients with MDBO (25–33).

A recent meta-analysis evaluating the outcomes of EUS-CDD with LAMS of 284 patients (of which the majority were patients with MDBO) across 7 studies demonstrated a pooled technical success rate of 95.7% (95% CI 93.2-98.1), clinical success rate of 95.9% (95% CI 92.8-98.9) and post procedure adverse event rate of 5.2% (95% CI 2.6-7.9).

Lastly, biliary decompression via drainage of the gallbladder in patients with a patent cystic duct could be considered if other forms of EUS-BD were not possible. This can be achieved by placement of a LAMS under EUS guidance draining the gallbladder into the duodenum or the stomach. Two recent studies of this technique in patients with MDBO have reported high technical and clinical success rates (34, 35).

#### 2.1.2 Safety of EUS-BD

Just like with any novel intervention, it would be expected that the safety and adverse event profile associated with EUS-BD evolves over time as more experience with the procedure is accrued and best practice is shared via collaborative work. Wang, et al. reported a systematic review of the safety of EUS -BD, comprising of 1192 patients over 42 studies (36). They demonstrated a cumulative adverse event rate of 23.32% which included events such as bleeding (4.03%), bile leaks (4.03%), pneumoperitoneum (3.02%), stent migration (2.68%), cholangitis (2.43%), abdominal pain (1.51%), and peritonitis (1.26%). The rate of pancreatitis was 0.5%, which represents a much lower rate than is expected from ERCP. Although uncommon, pancreatitis after EUS-BD could still occur if there was an immediate prior attempt at ERCP or if techniques which involve instrumental manipulation in the vicinity of the major papilla and pancreatic duct orifice were attempted.

One of the major limitations of the systematic review by Wang, et al. was the heterogenous study population due to their inclusion of the different EUS-BD techniques. A meta-analysis specifically evaluating the use of LAMS for EUS-CDD demonstrated a pooled adverse event rate of 5.6% amongst 284 patients from 7 studies, with the following occurrence of adverse events: bleeding (2.5%), perforation (1.5%), cholangitis (1.5%), bile leaks (1.2%) and abdominal pain (1.2%) (37). Crucially, they also demonstrated that the pooled rate of recurrent jaundice was 8.7% over the follow-up period with 90% of cases being due to obstruction of the lumen of the LAMS and the rest due to migration of the LAMS.

The duration of stent patency, recurrent jaundice and repeated cholangitis after LAMS insertion at EUS-CDD in patients with MDBO have been increasingly recognized to be a potential hinderance in causing delays in the patients’ pathway with interruptions to oncological treatment, repeated hospitalizations and compromise to their physiological reserves. It is hypothesized that stent dysfunction relating to reflux of enteric contents leads to blockage of the lumen of the LAMS. The risk of this is particularly amplified by the presence of gastric outlet obstruction from duodenal stenosis due to the increased volume of stagnant enteric contents (38). Vanella, et al. evaluated the risk of development of stent dysfunction of LAMS in 93 patients with MDBO and demonstrated a stent dysfunction rate of 31.8% after a mean follow-up period of 166 days (39). They also devised a unique classification of stent dysfunction demonstrating the various mechanisms in which patency of the LAMS could be compromised, and detailing the different endoscopic rescue strategies which was successful in the majority of patients.

#### 2.1.3 Comparison of EUS guided biliary drainage versus percutaneous biliary drainage

Since the inception of EUS-BD, it has challenged PTBD as the salvage procedure of choice in patients with MDBO after an unsuccessful ERCP. In 2012, Artifon, et al. performed the first randomized controlled trial (RCT) comparing both procedures (EUS-BD, n=13; PTBD, n=12) after unsuccessful ERCP with patients with MDBO (40). They demonstrated a 100% technical and clinical success rates in both groups, with a similar safety profile and cost effectiveness. Further RCTs have demonstrated there were a lower risk of adverse events and re-intervention rates in patients undergoing EUS-BD compared to PTBD (41, 42). These results are backed up by a number of meta-analyses reporting EUS-BD to be of similar efficacy to PTBD (43–45).

One of the major deficits in the literature relates to the relative dearth of comparator studies evaluating the QOL after both modalities. In the RCT by Lee, et al, they demonstrated no difference in QOL between both groups, despite a lower rate of adverse events and re-intervention rates in the PTBD group (41). A prospective, multi-centre trial designed as a non-inferiority trial to assess PTBD against EUS-BD in patients with MDBO after unsuccessful ERCP is underway, although interestingly the authors have not included QOL as an outcome measure in their trial design (46).

The other limiting factor in widespread uptake of EUS-BD is the fact that these procedures tend to only be available in tertiary hospitals and performed by expert endoscopists whereas PTBD is within the repertoire of most interventional radiologists. Local and regional networks play a part in determining which procedure a patient should be offered after a failed ERCP. Nevertheless, EUS-BD is widely recognized and recommended to be the salvage procedure of choice as long as it is feasible and there is available expertise (47).

#### 2.1.4 Comparison of EUS guided biliary drainage versus ERCP

ERCP remains the first-line modality for biliary decompression but can be unsuccessful in up to 15% of cases (14, 15). There are also associated risks of adverse events, particularly post-ERCP pancreatitis, with an incidence of up to 14% depending on underlying risk factors (48). The theoretical advantage of avoiding pancreatitis in patients undergoing EUS-BD as opposed to ERCP is also attractive.

Several studies have explored primary EUS-BD versus ERCP in patients with inoperable MDBO. Two RCTs by Bang, et al. and Paik, et al. demonstrated similar technical and clinical successes following EUS-BD and ERCP (49, 50). In both studies, EUS-BD was performed via a CDD route with the ‘traditional’ approach; entailing a multi-step procedure and subsequent placement of a tubular metal stent draining the bile duct into the duodenum. Similar results were demonstrated by another RCT by Paik, et al. who evaluated EUS-BD done via two different routes (‘traditional’ EUS-CDD with tubular metal stents and EUS-HG) (51). A meta-analysis of these RCTs (and including a fourth study, which was a retrospective cohort study) demonstrated similar efficacy and safety between both groups, but EUS-BD was associated with increased stent patency compared to ERCP (52).

It is crucial to point out that the above studies included only patients who had a ‘traditional’ EUS-CDD with tubular metal stents. Due to the increasing popularity and convenience of LAMS, most institutions have adopted this technique as the one of choice for EUS-CDD. The DRA-MBO RCT was recently published by Teoh, et al, being the seminal paper in the literature evaluating primary EUS-CDD with LAMS against ERCP in patients with inoperable MDBO. They demonstrated similar outcomes in clinical success, 30 day mortality, adverse events and 1 year stent patency rates between both groups (53). There was a stark difference between the technical success rates as EUS-CDD demonstrated a significant advantage over ERCP (96.2% vs 76.3%, p< 0.001). Even by exclusion of those patients who had an inaccessible papilla, there was still a 15% technical failure rate of ERCP, which illustrates the challenges of an ERCP in patients with MDBO and potentially strengthens the argument for primary EUS-CDD in patients with MDBO. However, generalized applicability of EUS-CDD could be hindered by anatomical factors. In the DRA-MBO study, an instance of technical failure occurred due to the presence of paraduodenal varices. An inadequately dilated bile duct could also be a relative contraindication with a diameter of 15mm typically suggested as a cut-off value (30). In essence, individualized decision making taking into account the patient’s anatomy as evaluated by pre-procedural cross sectional imaging is key. **Table 1** summarizes the key results of the current evidence base for RCTs evaluating EUS-BD versus ERCP.

**Table 1:** Key results of available randomized controlled trials in the literature evaluating endoscopic ultrasound guided biliary drainage versus endoscopic retrograde cholangiopancreatography for malignant distal biliary obstruction.

Authors	Year	Groups	Technical success	Clinical success	Adverse Events	Re-interventions
Bang*, et al*	2018	EUS-BD (n=33) ERCP (n=34)	90.9% 94.1% p=0.67	97% 91.2% p=0.61	21.2% 14.7% p=0.49	3.0% 2.9% p=0.99
Park*, et al*	2018	EUS-BD (n=15) ERCP (n=15)	93% 100% p=1.00	100% 93% p=1.00	0% 0%	*Not reported*
Paik, et al	2018	EUS-BD (n=64) ERCP (n=61)	93.8% 90.2% *Non-inferiority for EUS-BD reported*	90% 94.5% p=0.49	6.3% 19.7% p=0.03	15.6% 42.6% p=0.001
Teoh, et al	2023	EUS-BD (n=79) ERCP (n=76)	96.2% 76.3% p=<0.001	93.7% 90.8% p=0.559	16.5% 17.1% p=1.00	10.5% 12.1% p=0.48

#### 2.1.5 EUS guided biliary drainage in patients with operable disease

Up to 15-20% of patients with PDAC have disease that is operable at the time of diagnosis (54) and there is a rationale for pre-operative biliary decompression to restore the homeostatic mechanisms that is otherwise hampered by obstructive cholestasis. This includes, but is not limited to, the role of bile and bile acids in coagulation, immunoregulation and nutritional absorption (55, 56). The hypothetical benefit of improved surgical outcomes and overall survival after pre-operative biliary drainage remains debated with some evidence that avoiding pre-operative drainage may be more beneficial (57, 58). However, this may be influenced by the growing evidence of neo-adjuvant treatment, and the need for biliary drainage to facilitate this treatment, in patients with both borderline operable and operable disease (59). In the real world, there is no dogmatic approach towards pre-operative drainage and there is variation in practice, taking into account each individual patient’s condition, logistical scheduling and institutional preferences.

Real world data on pre-operative outcomes of ERCP was encapsulated by the Dutch Pancreatic Cancer audit. Latenstein, et al. demonstrated that 575 out of 1056 patients with resected pancreatic head or periampullary tumours underwent pre-operative endoscopic drainage, with an overall endoscopic related complication rate of 18.6% (pancreatitis in 8.2% and cholangitis in 7.5%) (60). The development of post-ERCP pancreatitis in particular could preclude successful surgical resection in patients who may have been deemed operable at the time of presentation due to deconditioning from the episode of pancreatitis, malignant progression of the disease whilst undergoing a period of convalescence, or creating a hostile surgical field at the point of operation rendering the resection technically impossible. As such, the benefit of EUS-BD over ERCP in these situations is the avoidance of pancreatitis (which would be the case if attempted ERCP was not performed beforehand). This would of course, have to be balanced with the other adverse events associated with EUS-BD, particularly bile leaks and biliary peritonitis (61), which is not associated with ERCP but may be circumvented by the usage of a LAMS due to its unique design.

There remains reticence regarding the applicability of EUS-BD in patients with operable disease and understandable concerns regarding subsequent surgical resection. There is increasing evidence that EUS-BD does not hinder subsequent pancreaticoduodenectomy, although this only applies to EUS-CDD as the choledochoduodenostomy tract and stent lies within the surgical resection field. This has been demonstrated by several studies (30, 32, 49, 62, 63).. Janet, et al. also included a comparator cohort group of patients who underwent ERCP and demonstrated a lower rate of post-operative complications in patients who had EUS-CDD (77.3% vs 93.7%, p=0.01) but no differences in the R0 resection rates, overall survival and progression free survival rates between both groups (63). With the caveat that this was a retrospective study, it adds to the body of evidence supporting EUS-CDD as a viable modality of biliary drainage in patients with operable disease, and instill the confidence in developing future RCTs comparing EUS-CDD against ERCP as a primary modality of biliary decompression in patients with resectable disease.

#### 2.1.6 Conclusion

The increasing body of evidence supporting the use of EUS-BD has led to it superseding PTBD as the salvage procedure of choice after unsuccessful ERCP in the European Society of Gastrointestinal Endoscopy (ESGE) guidelines (47) and Asia-Pacific expert consensus guidelines (64). Compared to EUS-BD, PTBD tends to be more readily available and not necessarily limited to tertiary instititutions, which is the case in the United Kingdom, although neither will be able to be provided out of hours at all times. Therefore, it is recommended that a tertiary institution creates arrangements within a regional network to provide an EUS-BD service for neighbouring ERCP providers. It must be said that there will still be a role for PTBD in patients with MDBO, for example in an acutely cholangitic or severely jaundiced patient where access to EUS BD is not available or if the procedure is not technically feasible.

The status quo of ERCP as the modality of choice for biliary decompression in patients with MDBO is also being challenged. This makes sense if the capabilities of EUS are maximally utilized in patients with MDBO; being able to obtain tissue, stage the disease and drain the biliary tract in one seating. The major limitation in affirming EUS-BD as the standard of care would be the fact that expertise is confined to tertiary institutions. Further studies are also required to identify specific cohorts of patients who would benefit most from primary EUS-BD.

### 2.2 EUS guided gastrojejunostomy

**Figure 3:**
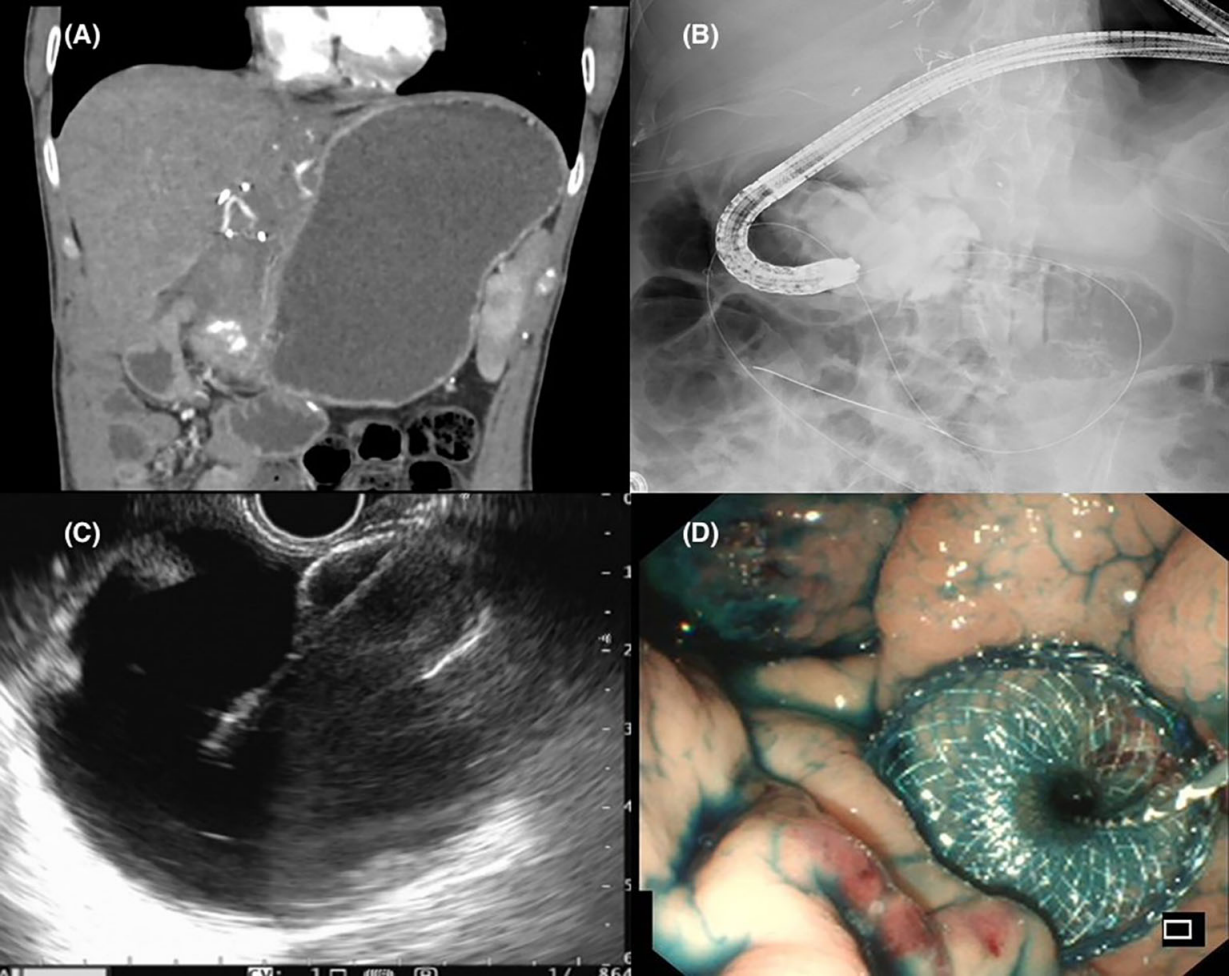
**(A)** Radiological evidence of gastric outlet obstruction. **(B)** Fluoroscopic capture of the stenosis being bypassed with a wire passed down an endoscope with contrast infused into the jejunum to identify a suitable limb for gastrojejunostomy formation. **(C)** Endosonographic views of a distended jejunal bowel loop and successful placement of a lumen apposing metal stent. **(D)** Endoscopic view following successful EUS guided gastrojejunostomy with reflux of methylene blue stained contrast solution into the stomach.

EUS guided gastrojejunostomy (EUS-GJ) was first reported by Binmoeller and Shah in 2012 (65). Although various techniques exist, the core premise is the endosonographic identification of a jejunal limb from the stomach and creation of a conduit between the stomach and the jejunum with the placement of a LAMS, thereby creating a preferential passage of food through this artificial conduit (66, 67). The key steps of EUS-GJ are summarized in **Figure 3**. Therefore, EUS-GJ confers a minimally invasive approach whilst placement of the stent at a distance away from the site obstruction, obviating the risk of tumour ingrowth into the stent, thereby reduces the chances of stent dysfunction and re-intervention rates.

#### 2.2.1 Gastric outlet obstruction

Gastric outlet obstruction (GOO) in patients with PDAC is characterized by localized tumoral infiltration of the distal stomach or duodenum, causing mechanical obstruction. Symptoms of GOO comprise early satiety, abdominal pain, nausea, vomiting, weight loss and failure to thrive (68). GOO may manifest at all stages of the disease course in PDAC and, if left untreated, will be a detriment to patients’ QOL and nutrition status, potentially rendering them unsuitable for surgical or oncological therapies (69). It should be noted that the majority of studies evaluating malignant GOO have a heterogenous study population by inclusion of patients with other malignancies in addition to those with PDAC, for example gastric cancer, duodenal cancer, and metastatic disease from other primaries.

Prior to the advent of interventional EUS, the mainstay of treatment in patients with malignant GOO was either endoscopic enteral stenting (ES) or a surgical gastrojejunostomy (S-GJ), via an open or laparoscopic approach (70, 71). ES has the benefit of being minimally invasive but its efficacy diminishes over time as GOO recurs when there is tumor ingrowth through the mesh of the stent or stent dysfunction occurs. On the contrary, S-GJ provides longer lasting patency to enable oral intake but is invasive and requires the patient to have a physiological and nutritional threshold to withstand an operative procedure (69).

Despite the relatively common incidence of malignant GOO, only three small RCTs exist with 27, 18 and 39 patients respectively. All three studies evaluated (72–74) ES against S-GJ, demonstrating shorter procedural time, quicker restoration of oral diet and shorter length of stay (LOS) in the ES group. However, the SUSTENT study demonstrated lower rates of re-intervention and longer lasting relief of GOO in the S-GJ group (74).

Since then, a large number of retrospective comparator studies have sought to compare various outcomes between ES and S-GJ. A recently published comprehensive meta-analysis compared 3,128 ES patients and 2,116 S-GJ patients across 39 studies (75). The authors demonstrated that the ES group had a shorter LOS, quicker restoration of oral diet and less surgical site infections. However, there was a greater risk of re-intervention (risk ratio 2.60, 95% Cl 1.87 to 3.63, p < 0.001), less likely to undergo adjuvant palliative chemotherapy (risk ratio 0.81, 95% Cl 0.70 to 0.93, p = 0.004) and a shorter survival time (mean difference -24.77 days, 95% Cl − 45.11 to  − 4.43, p = 0.02) in the ES group compared to the S-GJ group. The difference in survival time and commencement of palliative chemotherapy may be explained by the selection bias in patients undergoing ES, with these patients being more likely to have a poorer prognosis compared to patients in the S-GJ group. This point was succinctly expressed in an editorial by Adler (76) and it remains a limitation in drawing definite conclusions from retrospective studies, although it is clear that both ES and S-GJ remain viable options depending on the individual patient’s circumstances. S-GJ could be considered ahead of ES in patients with a good performance status and a life expectancy of over 3-6 months (77). It has to be noted that oncological advances have led to improved life expectancy in such patients. In patients who underwent ES successfully with sustained improvements in their nutritional status and are able to withstand a subsequent chemotherapy regimen, their prognosis is likely to exceed the initial expectations and it is this cohort of patients who may encounter stent dysfunction as time passes. Therefore, ES should be considered in patients who clearly have a prognosis which can be measured in weeks or short months.

#### 2.2.2 Safety of EUS guided gastrojejunostomy

EUS-GJ is firmly placed in the highest echelons of an interventional endoscopist’s skillset, and there is evidence that even an expert endoscopist has to scale a learning curve before achieving proficiency (78). It involves multiple steps, with little margin for error and requires significant technical and cognitive expertise to rectify errors, should they occur. Two meta-analyses have demonstrated the overall adverse event rate of 10-12%, including events such as bleeding, peritonitis, abdominal pain and stent misdeployments (79, 80).

A large retrospective review of 467 procedures from 12 tertiary institutions demonstrated a stent misdeployment rate of 9.85% (81). Although most were classed as mild to moderate, which could be managed endoscopically, there was a surgical intervention required in approximately 11% of cases. Therefore, if rescue surgery is required, this is likely to be of high risk due to the fact that these patients have an impaired physiological reserve resulting from their underlying disease state and a degree of malnutrition.

#### 2.2.3 EUS guided gastrojejunostomy vs endoscopic enteral stenting and surgical gastrojejunostomy

To date, there has not been any prospective RCT evaluating EUS-GJ against ES although there are a plethora of retrospective studies comparing both modalities (82–87). In all studies, the technical success of EUS-GJ was comparable to ES. In terms of clinical success, the results are less clear cut with some evidence to suggest that EUS-GJ is advantageous over ES (84, 85, 87). Of note, the reporting of clinical success and adverse events was variable across the studies with a mixture of definitions used in the literature. The study by Jaruvongvanich, et al. reported a significantly higher rate of adverse events in the ES group (38.9% vs 8.6%) although the majority of these events were related to stent obstruction or tumour ingrowth rather than procedural related adverse events (85). As would be expected, re-intervention rates were lower in patients who had an EUS-GJ (83–85, 87).

With regards to S-GJ in comparison with EUS-GJ, all studies are retrospective (82, 85, 88–93). In general, both technical and clinical success were similar between the groups. The inclusion of both open and laparoscopic approaches for S-GJ added additional heterogeneity, which may also account for higher adverse events in some studies, including bleeding, infection, anastomotic breakdown and ileus (85, 88, 89, 92). Crucially, the studies by Kouanda, et al. and Abbas, et al. demonstrated a shorter time to starting oncological treatment in the EUS-GJ group (88, 91). The study by Pawa, et al. showed that patients who underwent EUS-GJ had a shorter length of stay (4.3 vs. 8.2 days, p = 0.0009) and resumed oral diet quicker (1.0 vs. 5.8 days, p < 0.0001) compared to those who had a S-GJ via a robotic approach.

A meta-analysis concluded that clinical efficacy was of equal parity between all three modalities with similar safety profiles. Procedure related bleeding was least common but re-intervention rate was most common in the ES group (94). Although EUS-GJ has become increasingly popular, until the results of prospective studies are carried out, it is difficult to draw any firm comparisons. The American Society of Gastrointestinal Endoscopy (ASGE) guidelines for management of GOO published in 2021 acknowledges EUS-GJ within the evidence base but has not made any recommendations for its application (95). The ESGE guidelines for interventional EUS published in 2022 recommend EUS-GE as an alternative to ES or S-GJ but stop short of making specific criteria for choosing one over the other (47).

#### 2.2.4 Conclusion

The advent of interventional EUS and EUS-GJ has expanded the repertoire of procedures that are available for treating GOO. Although EUS-GJ is a promising technique, both ES and S-GJ are ‘tried and tested’ over the passage of time and both remain valid options for treating patients with GOO. It is likely that EUS-GJ and S-GJ would be on par in terms of providing symptomatic benefit for patients in the longer term with EUS-GJ having the advantage of being minimally invasive with a shorter recovery period. However, the practice of EUS-GJ is confined to specialist centres and performed by expert endoscopists. Ultimately, each modality has its merits and the decision making has to be individualized to the specific patient’s clinical condition, with life expectancy and physiological state taken into account of. The importance of multi-discplinary team (MDT) decision making is key.

### 2.3 EUS guided coeliac plexus intervention

Abdominal pain in patients with PDAC can range from mild to severe and debilitating, leading to a significant detrimental impact on QOL. It is highly prevalent, especially in patients with a primary tumor site in the body or tail of pancreas (96). More severe pain in patients with PDAC appears to be associated with worse performance status scores, when measured with the Eastern Cooperative Oncology Group (ECOG) and Karnofsky performance status scores (97). Interestingly, the pre-operative pain score also appears to be related to survival after resectional surgery. An observational study categorized 139 patients into three pain groups (none, mild, moderate-severe) based on a composite score evaluating pain severity, intensity and frequency. The median survival time after resection was 21.8 months, 15.0 months and 10.0 months (p=0.0015) in patients with no pain, mild pain and moderate-severe pain respectively (98). It is hypothesized that severity of pain may reflect a more advanced stage of disease at a microscopic level of neural invasion and likely also has collateral impact on patient related factors such as nutritional status and ability to tolerate oncological treatment (99).

#### 2.3.1 Pathophysiology of pain in PDAC

Pain in patients with PDAC share similar characteristics and mechanisms of action in patients with chronic pancreatitis and it is vital to commit research into the pathophysiology of pain in both groups of patients. The key concept is the neuroanatomy, which is characterized by the bidirectional pathways between the pancreas and the cerebral cortex, with the gland itself innervated by both sympathetic and parasympathetic nerve fibers of the autonomic nervous system (100). The coeliac plexus is one of the key gateways in this neural highway as it receives impulses via afferent neurons from the pancreas.

There are two main proposed mechanisms leading to the development of pain in patients with PDAC. The first mechanism relates to obstruction of the main pancreatic duct, impairing secretion of digestive enzymes into the duodenum and results in ductal and interstitial hypertension. This then impedes parenchymal blood flow and generates a pain of ischaemic aetiology, akin to a form of compartment syndrome (101). The second mechanism relates to development of a neuropathy due to a combination of factors including local activation of an inflammatory cascade from malignant cells that are present, direct malignant invasion of the perineurium, expression of cations involved in nociception such as the transient receptor potential cation channel and lastly, secretion of molecules that stimulate the nociceptive pathway (98, 99, 102, 103).

#### 2.3.2 Strategies to manage pain in PDAC

The optimum approach to improve pain in patients with PDAC often requires multiple modalities and also the involvement of the multi-disciplinary team. The initial method, and most convenient, of choice is with oral analgesics, which include the use of non-steroidal anti-inflammatories, opioids and neuroanalgesics. Strategies to escalate and individualize oral analgesics in the specific context of patients with PDAC are described by the National Comprehensive Cancer Network (NCCN) (104).

Chemotherapy itself could also have a beneficial effect on pain in patients with advanced PDAC. Kristensen, et al. conducted a systematic review of 30 studies investigating the impact of chemotherapy on QOL and performed a sub-analysis on 24 studies, which included pain scores as an outcome measure (105). They demonstrated that there was an improvement in pain with the delivery of chemotherapy, particularly with gemcitabine. There are of course, other systemic side effects relating to the use of chemotherapy and whilst it would not be expected that chemotherapy is commenced for the purposes of pain control, it may provide an additive analgesic benefit when given for oncological purposes. In addition, stereotactic body radiotherapy is another facet of oncological treatment which could improve pain control in patients with PDAC (106).

Finally, direct intervention to the coeliac plexus to manage pain can also be performed via an endoscopic or percutaneous route. It is important to recognize that the aforementioned different strategies to manage pain can be complementary and all avenues should be explored to achieve the best possible outcome for these patients.

#### 2.3.3 EUS guided coeliac plexus intervention

**Figure 4:**
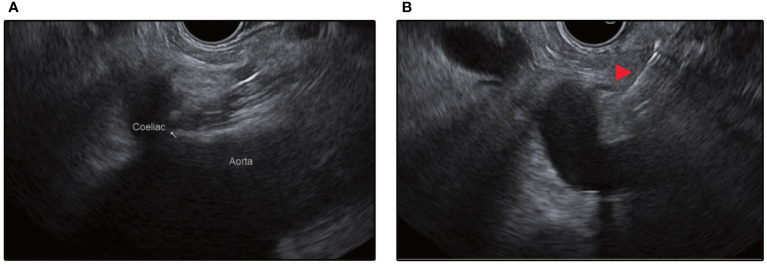
**(A)** Endosonographic identification of the aorta and the coeliac trunk take-off from the stomach. **(B)** Red arrow depicts injection needle targeting the space above the coeliac trunk for central injection.

Directed EUS guided therapy to the coeliac plexus (**Figure 4**) can be broadly divided into coeliac plexus neurolysis (CPN) or coeliac plexus block (CPB), depending on the injectable agent used. In CPN, typically ethanol or phenol is used whereas in CPB, combined steroids and local analgesia (such as triamcinolone and bupivacaine) are administered (107). CPB tends to be favoured in patients with pain from benign pancreatic disease as the use of ethanol in CPN leads to a localized inflammatory process, with subsequent fibrosis, which may hinder surgery if it were to be contemplated in these patients (108). Pain relief in these patients (either from CPN or CPB) tends not to last beyond 2-3 months. It is hypothesized that this is because the solvent flows away from its injected site due to its fluidity (109).

In 1996, Wieserma and Wieserma first reported the use of EUS CPN in a series of 30 patients with intraabdominal malignancies of whom 25 had underlying PDAC (110). They demonstrated that up to 88% of patients had an improvement in their pain scores at 12 weeks. Since then, several variations of EUS-CPN have been described (111, 112). The injection solvent can be injected either directly above the root of the coeliac artery (central injection) or either side of it (bilateral injections). The site of injection may also vary – coeliac ganglia neurolysis (CGN), which involves directly targeting the coeliac plexus ganglia or broad plexus neurolysis (BPN), which involves targeting the superior mesenteric ganglia.

#### 2.3.4 Efficacy and safety of EUS guided coeliac plexus intervention

Overall, studies have reported moderate to high efficacy of EUS guided coeliac plexus interventions in improving pain control in patients with PDAC. Multiple meta-analyses have reported improvement in pain scores after EUS-CPN in 70-80% of patients with PDAC (113–115). In their meta-analysis, Lu, et al. reported that patients with PDAC had similar improvements in short term pain relief after EUS-CPN regardless of whether a central or bilateral injection technique was performed although the bilateral technique led to a significant reduction in post-procedural analgesic use [RR = 0.66, 95% CI (0.47, 0.94), p = 0.02] (116).

Although EUS-CPN/B is considered a minimally invasive intervention, patients and clinicians have to remain vigilant of potential adverse events. Alvarez-Sánchez, et al. performed an analysis of 20 studies comprising 1,142 patients who underwent EUS-CPN/B, demonstrating that complications occurred in 7% of patients who had EUS-CPB (n=481) and 21% of patients who had EUS-CPN (n=661) (117). The majority of adverse events in both groups related to the injected solvent’s antagonistic impact on the sympathetic activity of the coeliac plexus and resultant unopposed parasympathetic activity, leading to diarrhoea and/or hypotension. Transient increase in pain post-procedurally can also be expected and is usually managed conservatively. Major complications occurred in 0.6% of patients after EUS-CPB (two patients developed abscesses and one developed a gastric haematoma) and 0.2% of patients after EUS-CPN (one patient developed retroperitoneal bleeding). There are also a handful of individual case reports describing the development of ischaemic multi-visceral injuries and development of paraplegia after EUS-CPN (117).

#### 2.3.5 Comparison with other modalities

To date, there have not been any RCTs evaluating EUS-CPN against percutaneous CPN in patients with PDAC although this has been studied in patients with chronic pancreatitis. The only published RCT was conducted by Wyse, et al. (118) They sought to evaluate the early use of EUS-CPN in patients who require an EUS for tissue acquisition of suspected PDAC compared to conventional pain management. Patients were randomized only after cytopathological analysis of the aspirate confirmed malignancy and the patient being deemed inoperable following strict criteria. The authors demonstrated that early EUS-CPN in patients with inoperable PDAC led to lower pain scores at 1 and 3 months, and a non-significant trend towards lower consumption of morphine.

With a distinct lack of comparative studies, it is therefore unsurprising that the position of EUS CPN in the algorithm of pain management in patients with PDAC is not well defined. The NCCN has no specific recommendations regarding the timing or route of coeliac plexus interventions but has advocated its usage in patients with unsatisfactory pain control and a high burden of analgesia (particularly opioid) usage (104). Similarly, the European Society for Medical Oncology recommends the use of CPN (favoring an endoscopic route over the percutaneous route) in patients with refractory pain and in those who are not in a poor clinical condition (119).

#### 2.3.6 Conclusion

Although EUS-CPN has been adopted in widespread practice for over a decade in patients with PDAC, there remains potential for ongoing research into the role of this in the treatment algorithm. There has not been direct comparison with other modalities such as percutaneous CPN, stereotactic radiotherapy or the plethora of analgesics that are available, including regimens that exclude opioid use. There is also variation in practice with at least three widely used approaches (CPN, CGN and BPN), and although all have been reported with similar efficacies in the literature, standardization of practice would require further comparator studies of all three techniques. Alternative neurolytic agents should also be studied to identify those that have the potential to provide a longer lasting analgesic effect.

### 2.4 EUS guided fiducial placement

Radiotherapy in PDAC is usually used for consolidation therapy for patients who have progressed despite first-line chemotherapy after 2-6 months or did not tolerate chemotherapy either with gemcitabine/nab-paclitaxel or FOLFIRINOX (120). Image-guided radiation and stereotactic body radiation therapy is increasingly being offered due to the shorter treatment duration and acceptable toxicity risk with a higher dose of radiation (121).

Fiducials are inert, radio-opaque markers that are placed into or near a target lesion to allow real-time tracking of the lesion. They are made from platinum or gold, creating low CT and MR artefacts while maintaining good visibility (120). They facilitate the delivery of higher doses of radiation and limit exposure of surrounding healthy tissue by quantifying tumor extent. Due to the implantation into the target tissue, fiducial markers may improve the localization and targeting of the lesion in comparison to using adjacent bony anatomy alone (122).

Pancreatic fiducial markers have been traditionally placed percutaneously under radiological guidance; however, there are concerns about the adverse event rates, including bleeding. Traditional methods of placement also carry increased rates of fiducial migration (123).

EUS-guided placement may be a more precise method to facilitate closer placements in or adjacent to the target tissue. It has high technical success of up to 92% with low rates of migration (124, 125). The fiducials can be delivered through preloaded needles or hand-loaded devices via different sized needles (126). EUS-guided placement involves the placement of at least three markers in different EUS planes and into the tumor at the periphery (120, 124). There is a 5- 8% risk of adverse events, including acute pancreatitis, cholangitis, bleeding, fever, and biliary stent migration (120, 124, 127).

Although pancreatic tumors are radiation resistant, the organs that lie in relation to it have increased radiation sensitivity (120). In addition to placing the fiducials, endoscopic assessment for duodenal involvement of the tumor prior to treatment can also aid in guiding the dosage of radiation needed.

Patients with PDAC can undergo biliary stenting to relieve malignant obstruction. Metal stents have been proposed as an alternative to fiducial placement to guide therapy. Although they may present better anatomical markers than traditional bony alignment, they still have larger margins for targeting compared to fiducials (128).

Care should be taken to place fiducials after tissue acquisition has been performed and a diagnosis and management plan for the patient has been discussed in MDT. The role of the MDT is critical in defining the diagnostic and therapeutic path and weighing in on the timing of fiducial placement. Placement of fiducial markers after commencing chemotherapy can be challenging due to desmoplastic reaction making the borders less well defined and the tumour hard (120). At this point, there is still insufficient evidence that placement of fiducial markers leads to improved outcomes from radiotherapy compared to conventional methods of radiotherapy planning with cross sectional imaging or other methods of fiducial markers placement. Performing an EUS for the sole indication of fiducial placements has to take into account the aforementioned risk of adverse events, which may preclude subsequent oncological treatment. Further prospective comparator studies evaluating EUS- guided fiducial placement are required to evaluate its impact on relevant patient outcomes.

### 2.5 EUS guided intratumoral therapy

As with fiducial markers, EUS allows precisely delivered intratumoral therapies. There is a clear advantage in the real time capability of assessing the effect of the therapy on the lesion itself via its endosonographic appearances to ensure complete and adequate treatment via a minimally invasive route. It is also possible to achieve a high level of localized drug concentration when injected directly into the tumor, which may be of advantage if systemic administration of the drug is limited by its potential toxicities. However, despite the recognition of the capability of EUS in administering direct tumoral therapy with precision, there is a relative paucity of studies evaluating this technique.

#### 2.5.1 Chemotherapy

In 2007, Matthes, et al. described EUS guided injection of paclitaxel into porcine pancreas with a demonstrable and sustained localized concentration of the drug up to 14 days post injection (129). Levy, et al. performed a study evaluating EUS guided injection of gemcitabine in 36 patients with locally advanced or metastatic PDAC (130). No adverse events relating to the EUS procedure were encountered. All patients then subsequently received either chemotherapy or chemoradiotherapy with a regime determined most appropriate by an oncologist. Interestingly, 4 out of 20 patients who were initially deemed unresectable were downstaged following treatment and each underwent an R0 resection. Whilst the nature of the study design precludes any specific impact intratumoral gemcitabine had on the subsequent resectability, at the very least, it demonstrates an avenue for further research.

#### 2.5.2 Immunotherapy

Unlike in other solid organ malignancies, the administration of systemic or localized immunotherapy has yet to take hold as a widely accepted modality of treatment in the oncological armamentarium in patients with PDAC. In a phase I clinical trial, cytoimplant (allogeneic mixed lymphocyte culture) was injected in 8 patients with unresectable PDAC, with an aim to upregulate host anti-tumor mechanisms via a local cytokine release (131). Despite modest efficacy as measured by tumor response on imaging (3 patients displayed response), a subsequent randomized trial comparing cytoimplant with gemcitabine suggested a worse outcome in patients who had cytoimplant, leading to termination of the trial.

Other intratumoral immunotherapies such as dendritic cell vaccines and oncolytic viruses have been studied. Dendritic cells function as antigen presenting cells to stimulate the host primary T-cell response and induce tumor antigen specific T lymphocytes with cytotoxic properties (132). Oncolytic viruses are considered a form of virotherapy whereby selective genetic engineering is carried out to enhance their affinity to specific tumors to induce oncolysis (133). These therapies have been the subject of small scale clinical studies and phase I/II trials. Herman, et al. reported a phase III RCT involving 304 patients with locally advanced PDAC evaluating TNFerade (an adenoviral vector capable of selective delivery of tumor necrosis factor-α) in combination with chemoradiation versus chemoradiation alone (134). There were no differences in survival between both groups.

### 2.6 EUS guided ablative therapies

Several modalities of ablative therapies have been demonstrated to be feasibly delivered via EUS with radiofrequency ablation (RFA) being the most prominent, although other forms such as photodynamic therapy, ethanol and laser ablation exist. The majority of the literature on EUS-RFA in pancreatic lesions lies within the study of this modality for pancreatic cystic lesions or pancreatic neuroendocrine tumors, but its utility in treating patients with PDAC is becoming increasingly studied.

The initial application of RFA in PDAC was via a peri-operative surgical approach during laparotomy as demonstrated in a prospective study of 50 patients with locally advanced PDAC by Girelli, et al. (135) In their study, there was a 24% rate of intra-abdominal adverse events that were attributable to RFA therapy. Crinò, et al. reported a feasibility study of EUS-RFA in 9 patients (8 of whom had PDAC), demonstrating that it was possible to achieve an ablation zone within the tumor margins and with no major adverse events (136). Similar conclusions were achieved by two separate studies involving only patients with PDAC; one with 6 patients by Song, et al. (137) and another with 10 patients by Scopelliti, et al. (138)

Overall, EUS-RFA appears to be fairly safe with a recent meta-analysis of 115 patients (with various pancreatic lesions being treated) demonstrating a pooled adverse event rate of 6.7% (95% CI: 3.4–11.7, I2 = 34.0%), the commonest being acute pancreatitis (3.3%) (139).

Photodynamic therapy (PDT) is a method of localized treatment of tumorous cells via administration of a photosensitizing agent which is activated by light leading to free oxygen radicals formation. Chan, et al. demonstrated in their pilot study involving porcine models that PDT could be feasibly delivered via EUS to induce a local ablation zone in the pancreas (140). Small studies of the use of EUS-PDT in humans with PDAC have demonstrated that a zone of necrosis can be safely achieved as detected on post procedural computed tomography scanning (141, 142).

There is yet to be standardization of the RFA or PDT technique with marked heterogeneity in reported studies, and how this may affect the fine balance between achieving efficacy and mitigating adverse events. Furthermore, all the studies on PDAC thus far have focused on technical feasibility of the procedure but other outcome measures such as symptom benefit, QOL and survival have yet to be studied. The functional outcome of ‘successful’ ablation as determined by endosonographic or radiological interpretation is difficult to quantify. Lastly, there has not been a uniformly acknowledged indication for EUS-RFA in patients with PDAC to determine who would best benefit from this intervention and how it fares against standard of care. Nevertheless, it appears to be a promising avenue of intervention and should be explored further with prospective studies.

## 3 Discussion

The field of interventional EUS has expanded rapidly over the past decade leading to substantial enthusiasm for its use in a variety of circumstances. Nonetheless, based on the review of the literature, we have the following observations.

Firstly, there is a lack of RCTs which is largely due to the relative novelty of interventional EUS as compared to other more widely accepted modalities of care. Although multiple meta-analyses on different aspects of interventional EUS have been published, there is general acknowledgement that the paucity of prospective data is a major limitation. This is likely to be amplified by the fact that interventional EUS continues to evolve and there is yet to be standardization of the plethora of techniques that have been described. There is also a general acceptance that a steep learning curve is associated with these procedures even amongst expert endoscopists (23, 78, 143). This is particularly pertinent in procedures such as EUS-BD and EUS-GJ, which consist of multiple steps with the potential for adverse event at each step, requiring technical and mental skill to rectify. It is only natural that innovation leads to collaborative working from which cumulative experiences across the endoscopy community worldwide have led to ongoing refinement of techniques and equipment.

Secondly, the majority of the studies have included patients with different aetiologies of MDBO or intraabdominal malignancies. Whilst PDAC tends to remain one of the majority patient groups, it is nonetheless a heterogenous study population. As each individual tumour biology and aggression differs, this is likely to affect the disease course and outcomes over the follow-up period.

Thirdly, the majority of studies have focused their primary outcomes on technical aspects such as procedural success and adverse event rates. Undoubtedly, these are relevant and important outcomes in the initial evaluation of an innovative technique. As experience and comfort with interventional EUS grows, there should be an impetus to evaluate more relevant outcomes such as survival, patient reported outcome measures, QOL, ability to receive anti-cancer treatment, and perhaps even composite endpoints.

Fourthly, there is heterogeneity of how key outcomes are defined and reported in the literature body of interventional EUS. These include parameters such as technical success, clinical success, adverse events, amongst others. The coming years are likely to see a multitude of prospective studies evaluating EUS-BD and it seems that the time is right for outcomes of interventional EUS to be formally defined.

In conclusion, interventional EUS has numerous applications in treating the multitude of clinical sequelae in patients with PDAC and should be considered where appropriate. Although interventional EUS has now established itself in the management algorithm of patients with PDAC (particularly EUS-BD and EUS-GJ), there remain numerous avenues for prospective studies that should be undertaken.

## Author contributions

WO conceptualized the idea, performed the literature review and drafted the manuscript. WA performed the literature review and drafted the manuscript. SE, MH and BP critically reviewed the manuscript. All authors contributed to the article and approved the submitted version.
